# Cannabis use and its socio-demographic correlates among in-school adolescents in Zambia

**DOI:** 10.1186/1824-7288-39-13

**Published:** 2013-02-15

**Authors:** Seter Siziya, Adamson S Muula, Chola Besa, Olusegun Babaniyi, Peter Songolo, Njinga Kankiza, Emmanuel Rudatsikira

**Affiliations:** 1 Department of Clinical Sciences, School of Medicine, Copperbelt University, Ndola, Zambia; 2 Department of Community Health, College of Medicine, University of Malawi, Blantyre, Malawi; 3 World Health Organization Country Office, Lusaka, Zambia; 4 School of Health Professionals, Andrews University, Berrien Springs, Michigan, USA

**Keywords:** Prevalence, Cannabis use, In-school, Adolescents, Zambia

## Abstract

**Background:**

Cannabis dependence in adolescents predicts increased risks of using other illicit drugs, poor academic performance and reporting psychotic symptoms. The prevalence of cannabis use was estimated two decades ago in Zambia among secondary school students. There are no recent estimates of the extent of the problem; further, correlates for its use have not been documented in Zambia. The objective of study was to estimate the current prevalence of cannabis use and its socio-demographic correlates among in-school adolescents.

**Methods:**

We conducted secondary analysis of data that was obtained from the 2004 Zambia Global School-Based Health Survey. Logistic regression analysis was conducted to identify the socio-demographic factors associated with cannabis use.

**Results:**

A total of 2,257 adolescents participated in the survey of which 53.9% were females. The overall prevalence of self reported ever-used cannabis was 37.2% (34.5% among males and 39.5% among females). In multivariate analysis, males were 8% (AOR = 0.92; 95% CI [0.89, 0.95]) less likely to have ever smoked cannabis. Compared to adolescents aged 16 years or older, adolescents aged 14 years were 45% (AOR = 1.45; 95% CI [1.37, 1.55]) more likely, and those aged 15 years were 44% (AOR = 0.56; 95% CI [0.53, 0.60]) less likely to report to have ever smoked cannabis. Other factors that were significantly associated with cannabis use were history of having engaged in sexual intercourse (AOR = 2.55; 95% CI [2.46, 2.64]), alcohol use (AOR = 4.38; 95% CI [4.24, 4.53]), and having been bullied (AOR = 1.77; 95% CI [1.71, 1.83]). Adolescents who reported being supervised by parents during free time were less likely to have smoked cannabis (AOR = 0.92; 95% CI [0.88, 0.95]).

**Conclusions:**

The use of cannabis is prevalent among Zambian in-school adolescents. Efforts to prevent adolescents’ psychoactive drug use in Zambia should be designed considering the significant factors associated with drug use in the current study.

## Background

Use of cannabis results in mental and behavioral disorders: intoxication, harmful use, dependence and psychotic disorders [1]. Cannabis use among adolescents varies between countries and within countries in Africa. Eneh and Stanley [2] found that 26% of secondary school students in Port Harcourt, Nigeria, used cannabis. Meanwhile, Abdulkarim et al. [3] reported that 3.4% of students in Ilorin, Nigeria, currently used cannabis. In Zimbabwe, Khan and Arnott [4] found that 3.4% of students in rural secondary schools had ever used cannabis. Another study in Zimbabwe by Acuda and Eide [5] found that 6.2% of students in rural and urban secondary schools had ever used cannabis. In KwaZulu-Natal, South Africa, Taylor et al. [6] found that 16.9% of school pupils in high schools had ever used cannabis. Differences in definitions of prevalence of cannabis use may partly explain the observed variations of cannabis use prevalence in Africa. Definition of cannabis use varied from prevalence of cannabis use at the time of the survey, in the previous one month to the survey, to ever use cannabis.

Age and sex are common demographic factors reported to be associated with cannabis use. Patrick et al. [7] observed that 21% of boys and 11% of girls in the 8th grade, and 39% of boys and 28% of girls in the 9th grade had used cannabis. Higher proportions of boys than girls who had used cannabis have also been reported elsewhere. In Seychelles, Faeh et al. [8] reported prevalence of cannabis use of 17% among boys and 8% among girls. Reddy et al. [9] reported in South Africa prevalence of 14.3% and 5.1% of past month cannabis use in males and females, respectively. In secondary school-going students in rural and urban Zimbabwe, Acuda and Eide [5] reported that significantly more male (9.5%) than female (1.9%) students had ever used cannabis.

In relation to age, Faeh et al. [8] reported that prevalence of ever used cannabis was more common among adolescents aged 15–17 years than in the 11–13 year and 14 years age groups. Khan and Arnott [4] reported that use of cannabis increased with age among students in rural secondary schools in Zimbabwe. Acuda and Eide [5] also reported in Zimbabwe that cannabis use prevalence increased with age in urban and rural secondary schools. In South Africa, Reddy et al. [9] observed that cannabis use was positively related to age.

Cannabis use is also related to other factors. Cannabis dependence in adolescents predicts increased risks of using other illicit drugs, poor academic performance and reporting psychotic symptoms [10]. South African adolescents who were substance users were more likely to engage in sex with unknown partners [11], which is a risk factor for HIV and other sexually transmitted infections.

Two decades ago Haworth [12] estimated the prevalence of cannabis among school-going female adolescents at 10% in Zambia. We are unaware of any studies that have reported on socio-demographic correlates of history of cannabis use among in-school adolescents in Zambia. We therefore used the Global School-Based Health Survey data to estimate the 2004 prevalence and socio-demographic correlates of cannabis use in Zambia. We believe that the knowledge on prevalence of use and the associated factors will not only add to the literature on illicit drug use in developing nations, but will also inform public health policy makers and program planners on how best adolescent drug use may be prevented or its prevalence reduced.

## Methods

This study involved secondary analysis of data from the Zambia Global School-based Health Survey (GSHS) conducted in 2004. The GSHS developed by the World Health Organization (WHO) in collaboration with UNICEF, UNESCO, and UNAIDS with technical assistance from the Centers for Diseases Control and Prevention (CDC), Atlanta, Georgia, United States, aims to provide data on health and social behaviours among in-school adolescents. Of the 3021 adolescents who were eligible to participate in the survey, 2257 eventually participated, giving a response rate of 74.7%.

The GSHS used a two-stage probability sampling technique, in which schools were the primary sampling units. The schools were selected with a probability proportional to their enrolment size. In the second step of sampling, a systematic sample of classes expected to have the majority of the students between 13 and 15 years in the selected schools was obtained. The school grades meeting this selection criterion were Grades 7 to 10 with Grade 7 being primary and Grades 8 to 10 being secondary school grades. All students in the selected classes, regardless of age, were invited to participate. A self-completed questionnaire was used to collect data. Students completed the questionnaire within one class period and 12 trained research assistants supervised the process.

The outcome variable was lifetime cannabis use. Predictor variables included having had sexual intercourse within the last 12 months, alcohol consumption, gender, and adolescents’ own assessment of parental supervision. Some of the questions asked were: During the past 12 months, have you had sexual intercourse? (Yes/No). The main outcome was assessed through the question: During your life, how many times have you used dagga? Study participants who indicated at least one time of use were re-coded as 1, else 0. During the past 30 days, on how many days did you have at least one drink containing alcohol?

### Data analysis

Data analysis was performed using SPSS version 14.0 software. A weighting factor was used in the analysis to reflect the likelihood of sampling each student and to reduce bias by compensating for differing patterns of non response. We obtained frequencies as estimation of prevalence of the main outcome (having used cannabis) and other descriptive characteristics of the sample. Proportions were compared using the Yates’ corrected Chi-square test at a 5% significance level. We conducted backward logistic regression analysis to estimate the association between relevant predictor variables and self-reported history of having ever smoked cannabis. We report unadjusted odds ratios (OR) for selected predictor variables while considering having smoked as a dependent variable. We also report results for multivariate analysis (adjusted odds ratios - AOR) for the factors found significantly associated with the outcome in bivariate analysis. In addition 95% confidence intervals (CI) for the odds ratios are reported.

### Ethical considerations

We conducted the study using data that are de-identified and are in public domain with global authorization for re-use. Both the Ministries of Health and Education provided ethical oversight of the Zambia GSHS. Informed consent to participate in the study was collected from students aged 16 years or older; and parental consent was obtained for those younger than 16 years. The school managers gave permission to conduct the survey. Confidentiality was upheld by allowing for anonymity in completing the questionnaire.

## Results

A total of 2257 respondents participated in the survey of which 53.9% were females. Overall, 45.2% of the respondents (55.0% of males and 32.9% of females) reported to have ever had sexual intercourse. Meanwhile, 42.2% of the respondents (38.5% of males and 45.1% of females) had consumed alcohol. The overall prevalence of ever smoked cannabis was 37.2% (34.5% among males and 39.4% among females; p = 0.037). The rest of the description is shown in Table 1.

**Table 1:** Socio-demographic characteristics of adolescents in the Zambia global school-based health survey, 2004

	**Total**	**Males**	**Females**
**Factor**	n* (%)**	n* (%)**	n* (%)**
**Age**			
<13	463 (27.5)	156 (21.5)	263 (30.9)
14	386 (19.1)	156 (17.4)	219 (21.6)
15	513 (22.8)	260 (24.5)	238 (21.6)
16+	708 (30.6)	394 (36.5)	306 (25.9)
**Sex**			
Male	994 (46.1)	-	-
Female	1039 (53.9)	-	-
**Ever had sexual intercourse**			
Yes	303 (45.2)	178 (55.0)	111 (32.9)
No	387 (54.8)	145 (45.0)	232 (67.1)
**Consumed alcohol**			
Yes	528 (42.2)	217 (38.5)	283 (45.1)
No	805 (57.8)	388 (61.5)	388 (54.9)
**Bullied**			
Yes	949 (62.8)	431 (60.0)	466 (65.0)
No	610 (37.2)	314 (40.0)	274 (35.0)
**Parental supervision**			
Yes	1369 (77.4)	634 (78.1)	673 (77.2)
No	377 (22.6)	169 (21.9)	189 (22.8)
**Ever smoked dagga**			
Yes	695 (37.2)	300 (34.5)	357 (39.4)
No	1301 (62.8)	634 (65.5)	614 (60.6)

* unweighted frequencies ** weighted percents.

Table 2 shows results of bivariate and multivariate analyses. The results of the two analyses were similar, except for the respondents of ages younger than 14 years. While respondents younger than 14 years were more likely to ever smoked cannabis compared to those of ages 16 years or older in the bivariate analysis, there was no significant difference in the odds of ever smoked cannabis between the two age groups in the multivariate analysis.

**Table 2:** Factors associated with ever smoked dagga among in-school adolescents in Zambia

	**Bivariate analysis**	**Multivariate analysis**
**Factor**	**OR (95% CI)**	**OR (95% CI)**
**Age**		
<13	1.14 (1.12, 1.15)	1.03 (0.97, 1.09)
14	1.32 (1.30, 1.33)	1.45 (1.37, 1.55)
15	0.82 (0.81, 0.83)	0.56 (0.53, 0.60)
16+	1	1
**Sex**		
Male	0.90 (0.89, 0.91)	0.92 (0.89, 0.95)
Female	1	1
**Ever had sexual intercourse**		
Yes	2.59 (2.55, 2.63)	2.55 (2.46, 2.64)
No	1	1
**Consumed alcohol**		
Yes	4.06 (4.02, 4.11)	4.38 (4.24, 4.53)
No	1	1
**Bullied**		
Yes	2.46 (2.44, 2.49)	1.77 (1.71, 1.83)
No	1	1
**Parental supervision**		
Yes	0.96 (0.95, 0.97)	0.92 (0.88, 0.95)
No	1	1

In multivariate analysis, while respondents aged 14 years were 45% more likely to ever smoked cannabis than those aged 16 years or older, respondents of age 15 years were 44% less likely to ever smoked cannabis compared with respondents aged 16 years or older. Compared to female respondents, males were 8% (OR = 0.92; 95% CI [0.89, 0.95]) less likely to ever smoked cannabis. Respondents who ever had sexual intercourse were 2.55 (95% CI [2.46, 2.64]) times more likely to ever smoked cannabis compared to those who never had sexual intercourse. Compared with respondents who did not consume alcohol, those who consumed alcohol were 4.38 (95% CI [4.24, 4.53]) times more likely to ever smoked cannabis. Respondents who reported being bullied were 77% (OR = 1.77; 95% CI [1.71, 1.83]) more likely to ever smoked cannabis compared to those who did not report being bullied. Respondents who received parental supervision were 8% (OR = 0.92; 95% CI [0.88, 0.95]) less likely to ever smoked cannabis compared to those who did not receive the supervision.

## Discussion

We estimated an overall prevalence of having ever smoked cannabis at 37.2% among in-school adolescents in Zambia. Comparing our prevalence to those reported in the African region, we note that our prevalence is higher than the 26% reported in Port Harcourt in Nigeria [2] and the 16.9% reported in KwaZulu-Natal in South Africa [6], and much higher than that reported in Zimbabwe of 3.4% in rural schools [4] and 6.2% in both rural and urban schools [5]. As earlier stated, differences in definitions of prevalence of cannabis use may partly explain the observed variations of cannabis use prevalence in Africa.

Results on the association between gender and cannabis use has not been consistent. Ever use prevalence for cannabis for females was higher than that for males (39.4% versus 34.5%) in the current study. This is not in line with studies from elsewhere where cannabis smoking was more common among males compared to females [5,7-9]. While anecdotal evidence suggested that there was an increase in the proportion of secondary school-going girls who smoked cannabis, we did not think that the proportion of girls who used cannabis would be higher than that for boys. It appears it had become a fashion for girls to smoke, thus surpassing the prevalence for boys.

In the present study, adolescents aged 14 years or younger were more likely to have used cannabis compared to those 15 years or older. The following are plausible explanations for the observed association between age and cannabis use. It is possible that older adolescents may be underreporting or younger adolescents may be over-reporting use. It is also possible that adolescents who experiment with cannabis at younger age stop when they are older, hence the lower prevalence at higher age groups. Results from the current study contradicts those obtained from elsewhere in which it is reported that prevalence of cannabis use increases with age [4,5,8].

It is of interest that adolescents who reported having ever used cannabis also were more likely to report having used alcohol and engaged in sexual intercourse. We suggest these findings confirm the clustering harmful health lifestyles among adolescents reported in previous studies [8,13].

Adolescents who reported to have been bullied were also likely to have used cannabis before. Although we cannot assign causation in this cross sectional study, the finding potentially raises important issues. It is possible that adolescents who find themselves in situations that predispose them to bullying may also be in situations where cannabis use is likely. It is also possible that propensity to be bullied may be associated with use of cannabis. In a very large study involving 78,333 middle and high school students in United States, Radliff et al. [14] observed a link between involvement in bullying and substance abuse.

Our study has shown that adolescents who reported being supervised by parents in their free time were 8% less likely to have used cannabis. The role of the relationship between parental supervision and risk behaviors among adolescents has been reported before [13].

### Limitations of the current study

The present study had several limitations. Firstly, data were collected from self-reports. To the extent that respondents mis-reported due to not being able to recall inadvertently or intentionally, our findings would be biased. Data on self reports were not triangulated with interviews of significant others in the adolescent’s sphere of life. No biomarker studies were carried out among those who reported current cannabis use or any of the independent variables. There could have been unmeasured confounders which we did not control for in the multivariate analysis. About a quarter of the adolescents did not respond to the questionnaire. Our findings may not be generalizable to the entire in-school adolescents if those who did not respond differed in some characteristics from those who responded to the questionnaire. However, we do not have any information on non-respondents to allow us to compare their characteristics to those who responded.

## Conclusions

In a study of Zambian in-school adolescent, we found a lifetime prevalence of cannabis use of 37.2%. We also found that parental supervision and being male were associated with lower likelihood of reporting cannabis use. Having reported being bullied, alcohol and sexual intercourse were associated with history of having used cannabis. The present study also confirms the observations from other studies of the clustering of harmful lifestyles among adolescents. Interventions aimed at promoting health among adolescents may need to have this observation into consideration.

## Competing interests

Authors declare that they have no competing interests.

## Authors’ contributions

SS conducted the data analysis, participated in the interpretation of the results and drafting of the manuscript. ASM participated in the interpretation of the results led the drafting of the manuscript. CB, OB, PS, NK and ER participated in the interpretation of the data and drafting of the manuscript. All authors read and approved the final manuscript.

## References

[B1] WHO The world health report 2001. Mental health-new understanding, new hope 2001 Geneva: World Health Organization

[B2] Eneh AU Stanley PC Pattern of substance use among secondary school students in Rivers State Niger J Med 2004 13 36 39 15296105

[B3] Abdulkarim AA Mokuolu AO Adeniyi A Drug use among adolescents in Ilorin, Nigeria Trop Doct 2005 35 225 228 10.1258/004947505774938620 16354478

[B4] Khan N Arnott R Substance use among rural secondary schools in Zimbabwe: patterns and prevalence Cent Afr J Med 1996 42 223 229 8990565

[B5] Acuda SW Eide AH Epidemiological study of drug use in urban and rural secondary schools in Zimbabwe Cent Afr J Med 1994 40 207 212 7812998

[B6] Taylor M Jinabhai CC Naidoo K Kleinschmidt I Dlamini SB An epidemiological prospective of substance use among high school pupils in rural KwaZulu-Natal S Afr Med J 2003 93 136 140 12640886

[B7] Patrick ME Collins LM Smith E Caldwell L Flisher A Wegner L A Prospective Longitudinal Model of Substance use Onset among South African Adolescents Subst Use Misuse 2009 44 647 662 10.1080/10826080902810244 19360538 PMC2796627

[B8] Faeh D Viswanathan B Chiolero A Warren W Bovet P Clustering of smoking, alcohol drinking and cannabis use in adolescents in a rapidly developing country BMC Public Health 2006 6 169 10.1186/1471-2458-6-169 16803621 PMC1564395

[B9] Reddy P Resnicow K Omardien R Kambaran N Prevalence and correlates of substance use among high school students in South Africa and the United States Am J Public Health 2007 97 1859 1864 10.2105/AJPH.2006.086330 17761580 PMC1994193

[B10] Hall WD Cannabis use and the mental health of young people Aust N Z J Psychiatry 2006 4 105 113 16476127 10.1080/j.1440-1614.2006.01756.x

[B11] Palen LA Smith EA Flisher AJ Caldwell LL Mpofu E Substance use and sexual risk behaviours among South African eighth grade students J Adolesc Health 2006 39 761 763 10.1016/j.jadohealth.2006.04.016 17046518

[B12] Haworth A A preliminary report on self-reported drug use among students in Zambia Bull Narc 1982 34 45 60 6985027

[B13] Siziya S Muula AS Kazembe LN Rudatsikira E Harmful lifestyles’ clustering among sexually active in-school adolescents in Zambia BMC Pediatr 2008 8 6 10.1186/1471-2431-8-6 18267020 PMC2276492

[B14] Radliff KM Wheaton JE Robinson K Morris J Illuminating the relationship between bullying and substance use among middle and high school youth Addict Behav 2012 37 569 572 10.1016/j.addbeh.2012.01.001 22277772

